# C-952-06. Peripheral IV Placement and Longevity in the Pediatric Burn Population: Can Ultrasound Guidance Improve Practice?

**DOI:** 10.1093/jbcr/irag033.122

**Published:** 2026-04-06

**Authors:** Gordon Barcomb, Richard Brown, Stacye D Smith

**Affiliations:** Shriners Children's Texas, Galveston, TX; Shriners Children's Texas Burn Center, Galveston, TX; Shriners Children's Texas Burn Center, Galveston, TX

## Abstract

**Introduction:**

Peripheral intravenous (PIV) access is an essential component of nursing care but is often challenging in the pediatric burn population due to limited vascular access sites, smaller anatomy, and compromised skin integrity. Ultrasound-guided PIV (USGPIV) placement has been shown to improve outcomes in pediatric patients, but limited evidence exists in pediatric burn populations. We postulate that the use of ultrasound guidance for placing PIVs in pediatric burn survivors can reduce the number of insertion attempts and extend catheter life.

**Methods:**

A quality improvement project was implemented to assess the impact of USGPIV training on PIV placement. Twenty-three nurses completed structured didactic and hands-on training, followed by bedside coaching. Over seven months, data were collected on 83 inpatient PIV insertions, comparing traditional placement with USGPIV. Outcomes included number of attempts, catheter longevity, and duration of blood draw usability.

**Results:**

Of 83 PIVs, 55 were placed traditionally and 28 with ultrasound. Average attempts were 1.3 for traditional versus 1.5 for USGPIV. Catheter longevity improved from 2.3 days (traditional) to 3.5 days (ultrasound), a 52% increase. Duration of blood draw usability increased from 1.3 to 1.8 days, a 38% improvement. Documentation inconsistencies and selection bias likely influenced the attempts outcome.

**Conclusions:**

Although USGPIV did not reduce insertion attempts, it significantly improved catheter longevity for both infusion and blood collection in pediatric burn patients. Findings suggest USGPIV may reduce reliance on central venous catheters and enhance patient safety. Improved documentation and project design are recommended to better evaluate its full impact.

**Applicability of Research to Practice:**

Training nurses in USGPIV offers a practical strategy to improve vascular access in challenging populations, potentially decreasing central line use and associated complications while improving patient and caregiver satisfaction.

**Funding for the study:**

N/A.

